# Circulating C1q/TNF-Related Protein 3, Omentin-1 and NGAL in Obese Patients with Type 2 Diabetes During Insulin Therapy

**DOI:** 10.3390/jcm8060805

**Published:** 2019-06-05

**Authors:** Katarzyna Komosinska-Vassev, Pawel Olczyk, Kornelia Kuźnik-Trocha, Agnieszka Jura-Półtorak, Alicja Derkacz, Marcin Purchałka, Alicja Telega, Krystyna Olczyk

**Affiliations:** 1 Department of Clinical Chemistry and Laboratory Diagnostics, School of Pharmacy and Division of Laboratory Medicine in Sosnowiec, Medical University of Silesia, 41-200 Sosnowiec, Poland; kkuznik@sum.edu.pl (K.K.-T.); ajura@sum.edu.pl (A.J.-P.); derkacz.alicja@gmail.com (A.D.); marcin.purchalka@gmail.com (M.P.); alicjatelega@tlen.pl (A.T.); olczyk@sum.edu.pl (K.O.); 2 Department of Community Pharmacy, School of Pharmacy and Division of Laboratory Medicine in Sosnowiec, Medical University of Silesia, 41-200 Sosnowiec, Poland; polczyk@sum.edu.pl

**Keywords:** adipose tissue, adipocytokines, obesity, type 2 diabetes, omentin-1, NGAL, C1q/TNF-related protein-3 (CRTP3), insulin therapy

## Abstract

The aim of the study was to quantify the plasma concentration of omentin-1, neutrophil gelatinase-associated lipocalin (NGAL), and complement C1q tumor necrosis factor-related protein-3 (CTRP3) in obese patients with type 2 diabetes, before introducing insulin therapy, in relation to the plasma expression profiles of these regulatory molecules in the same patients after a 6-month insulin mixture therapy and in obese controls. Elevated plasma NGAL concentrations were found in type 2 diabetic patients as compared with subjects with metabolically healthy obesity. In turn, a 6-month insulin mixture therapy has shown a marked increase in the plasma concentration of omentine-1 and a significant decrease in plasma CTRP3 concentration in obese patients with type 2 diabetes, in relation to the values found in these patients before the implementation of insulin therapy. Insulin mixture therapy has also proved to be an important factor modifying the plasma profile of NGAL, increasing the concentration of this bioactive molecule in the plasma of patients with type 2 diabetes, after 6 months of its use, in relation to the concentration before treatment. The significant changes in the plasma profile of omentin-1, NGAL and CTRP3 during insulin therapy suggest their potential diagnostic utility in monitoring metabolic changes associated with the introduction of insulin treatment in type 2 diabetic patients.

## 1. Introduction

Type 2 diabetes (T2DM) belongs to the group of metabolic diseases, which are directly caused by a secretion disorder and/or abnormal insulin action—a key hormone in maintaining the energy homeostasis of the body. Currently, there is increasing morbidity of this type of diabetes, which is most likely associated with improper lifestyle, as well as epidemics of obesity [1,2]. Obesity is now considered to be a medical condition with a complex etiopathogenesis and a negative impact on the regulation of carbohydrate metabolism. Adipose tissue has been long perceived only in terms of its storage function. Now, it is known that it plays an important role as the main endocrine organ, being the source of many regulatory molecules called adipocytokines. These substances, based on the current state of knowledge, are considered to be a “link” between adipose tissue with metabolic disorders such as metabolic syndrome or diabetes. In obesity, accumulated fat tissue, depending on the current metabolic status, is characterized by a different secretory profile, leading to disruption of insulin sensitivity and metabolic disturbances [3,4,5,6]. Among the recently identified regulatory peptides secreted by both adipose tissue and peripheral organs, which play an important role in the pathogenesis of metabolic changes in the course of obesity associated with type 2 diabetes, omentin-1, neutrophil gelatinase-associated lipocalin and complement C1q tumor necrosis factor-related protein-3 (also known as cartonectin or cartducin), are an essential part [4,7,8]. The first of them, omentin-1, mainly produced in visceral fat tissue, belongs to adipokines with anti-inflammatory activity and is also considered as a molecule increasing insulin sensitivity. Apart from insulin and glucose, which decrease its concentration, factors that affect its expression, also include inflammation which stimulates the increase of its plasma concentration [4,9]. NGAL, which belongs to the family of lipocalin, participates in the regulation of the immune response, hence its biological role is still not fully understood. Currently, it is supposed that the protein is linked to the regulation of insulin resistance and may be involved in the pathogenesis of obesity [7]. To date, research has revealed an increase of the NGAL content in adipose tissue of obese patients with type 2 diabetes. Moreover, plasma concentration of NGAL was in direct relation to insulin resistance and hyperglycaemia in these patients [7]. NGAL is an important regulator of the PPARγ (peroxisome proliferator-activated receptors γ) activity which is involved in the control of adipogensis and lipogenesis in adipose tissue and liver. Thus, NGAL plays a crucial role in regulating the glycolipids’ and energetic metabolism [10]. The third of the assessed adipokines, cartonectin (CTR3) is a recently discovered protein produced mainly by mature adipocytes [8]. CTRP3 belongs to the family of the C1q/TNF protein, the common feature of which is a deep structural homology to adiponectin (ADPN). The latter is the best-known member of the CTRP family linked to the regulation of insulin sensitivity in tissues and an important and recognized biomarker of the evaluation of treatment effectiveness in obese patients with metabolic disorders [8,11]. The decrease of plasma concentration of this adipocytokine is strictly related to insulin resistance and type 2 DM development [8]. However, blocking ADPN expression in transgenic mice leads to varied and relatively mild metabolic disorders, which suggests the existence of other, additional regulatory molecules, such as CTRP3 which protect the organism against the effects of adiponectin deficiency and contribute to maintaining energetic homeostasis of the system [8,12]. According to recent studies, CTRP3 participates in inflammatory and immunological processes and by reducing the secretion of pro-inflammatory cytokines - IL-6 and TNF, shows anti-inflammatory activity [11,13,14]. The concentration of CTRP3 in serum is inversely correlated with the leptin concentration. Cartonectin inhibits the processes of glucogenesis and adipogenesis. It is also suggested that cartonectin may decrease lipotoxicity associated with abnormal fat deposition in peripheral tissues. CTRP3 promotes proliferation and migration of endothelial cells during angiogenesis and acts as a weak vasodilator [15]. It is likely that CTRP3 performs similar functions to adiponectin, i.e., it increases insulin sensitivity, and has anti-inflammatory and cardioprotective action [15,16].

Current research indicates that adipocytokines are involved not only in the pathogenesis of metabolic disorders associated with obesity, but are also used as markers to assess the therapy or to predict the development of diabetic complications. Considering the above, the primary objective of this study was to quantify the plasma concentration of selected regulatory molecules, such as omentin-1, NGAL and CTRP3 in the plasma of obese patients with type 2 diabetes, before introducing insulin therapy, in relation to the concentration of these molecules in plasma of obese subjects in the control group. A key element of the study was an attempt to assess the impact of a 6-month insulin mixture therapy on the plasma expression profile of the regulatory molecules. For a better assessment of metabolic changes in the course of type 2 diabetes, the relationship between plasma concentration of the adipocytokines and the body mass index was also determined together with the, carbohydrate-lipid metabolism parameters in obese patients in the control group and patients with type 2 diabetes before implementation of insulin therapy and after a 6-month treatment period.

## 2. Experimental Section

Blood samples were collected from 42 obese patients with T2DM, males and females, aged 50–70, diagnosed with type 2 diabetes, and with mean diabetes duration of 13 years. The exclusion criteria were: Patients with type 1 diabetes (T1DM) and with other types of diabetes; female patients with gestational diabetes and diabetes during lactation; patients with autoimmune diseases; patients treated with glycocorticosteroids, adrenocorticotropic hormone (ACTH), and interferons; subjects with a prior stroke or myocardial infarction; patients with unstable angina; patients with class III and class IV heart failures according to New York Heart Association (NYHA); patients with liver failure; subjects with kidney failure as well as patients with hyperthyroidism and other endocrine and metabolic diseases. The control material consisted of blood samples obtained from 20 age-matched healthy, obese subjects of both sexes. Subjects in the control group were screened by means of medical history, physical examination and laboratory analyses. Basic biochemical and hematological blood tests were performed on every person qualified for the examination. Anthropometric measurements of patients were carried out including, among others, BMI (body mass index) and WHR (waist and hips ratio) assessment. The exclusion criteria were the presence of medical or surgical illnesses (clinical evidence of inflammatory disease, diabetes mellitus, cancer or kidney and liver disease), hospital admissions during the previous 3 years, current smoking, alcohol abuse, treatment for hyperlipidemia, antihypertensive or vasoactive medication and non-steroidal anti-inflammatory drugs.

Venous blood obtained from controls and T2DM patients after overnight fasting was drawn into blood collection tubes containing additives and without the addition of an anticoagulant and allowed to clot at room temperature. After centrifugation at 1500× *g* for 5 min at 4 °C, plasma and serum were removed and used for biochemical analyses. The assessment of anthropometric parameters (BMI and WHR) and laboratory biochemical examinations, including plasma fasting glucose, HbA1c, triglyceride, cholesterol, high-density lipoprotein (HDL), low-density lipoprotein (LDL), creatinine, uric acid, alanine aminotransferase, TSH and CRP were performed. Plasma samples were stored at −20 °C until they were used for circulating adipocytokines (omentin-1, NGAL, CTR3) analysis. In patients with T2DM the examinations were carried out twice: At the beginning of the treatment and six months after the treatment. Patients were treated with a biphasic insulin mixture 70/30 twice daily at breakfast and dinner.

The clinical characteristics of the healthy subjects and diabetic patients are shown in Table 1.

During the entire investigation period, the guidelines and regulations of the Helsinki Declaration were used. The Bioethics Committee of the Medical University of Silesia in Katowice (KNW/022/KB1/147/10) approved the research protocol used in this study. Written informed consent was obtained from all healthy volunteers and T2DM patients enrolled in the study.

Plasma omentin-1 level was measured using immunoassay from BioVendor (Brno, Czech Republic) according to the manufacturer’s instructions. The analysis of all samples was completed in one day to eliminate the effects of inter-assay variation. The kit was highly sensitive and specific, as the minimum detectable limit was 0.5 ng/mL, and the average intra-assay coefficient of variation was below 5%. The quantitative measurement of plasma lipocalin-2 (NGAL) was determined using a sandwich enzyme-linked immunosorbent assay (ELISA) kit supplied by BioVendor (Brno, Czech Republic). The limit of detection was 2.5 ng/mL and the intra-assay CV that quantified variation in the assay technique itself was less than 7.5 %. The plasma cartonectin (CTRP3) levels were measured by using immunoassay from Cloud-Clone Corp. (Houston, TX, USA) according to the directions provided by the manufacturer. The cartonectin levels were recorded as ng/ml. The limit of detection was 0.156 ng/ml. The intra-assay coefficient of variation (CV) was less than 8.5%.

Data analyses were performed using the StatSoft, Inc. (2014), STATISTICA, Tulsa, OK, USA (data analysis software system), version 12. (https://www.statsoft.pl). The normality of the distribution was verified using the Shapiro-Wilk test. The homogeneity of variance was assessed by Levene’a test. Variables are summarized as mean ± SD. Student’s t-test was used to determine whether the differences between means for patients and controls were significant. *p* Values of less than 0.05 were considered significant. To compare the same parameters in each patient before the treatment and after a 6-month insulin therapy period, a paired Student’s t-test was used. Pearson correlation coefficient was used to evaluate correlation between plasma adipocytokines and the body mass index, and carbohydrate-lipid metabolism parameters in obese patients with T2DM.

## 3. Results

There was no significant difference as regards age, BMI, and WHR among the studied groups. Fasting blood glucose and HbA1c values were higher in obese type 2 diabetic patients before insulin implementation compared to the control group. In diabetic patients after 6 months with the insulin mixture therapy, a significant increase in HbA1c, triglycerides and uric acid was observed compared to the concentration of these parameters in these patients before treatment. No significant differences were found in other metabolic indices between diabetic groups (Table 1).

A report on plasma omentin-1, NGAL and CTRP3 level in control subjects and diabetic patients before and after a six-month therapy with insulin is given in Table 2.

**Figure 1:**
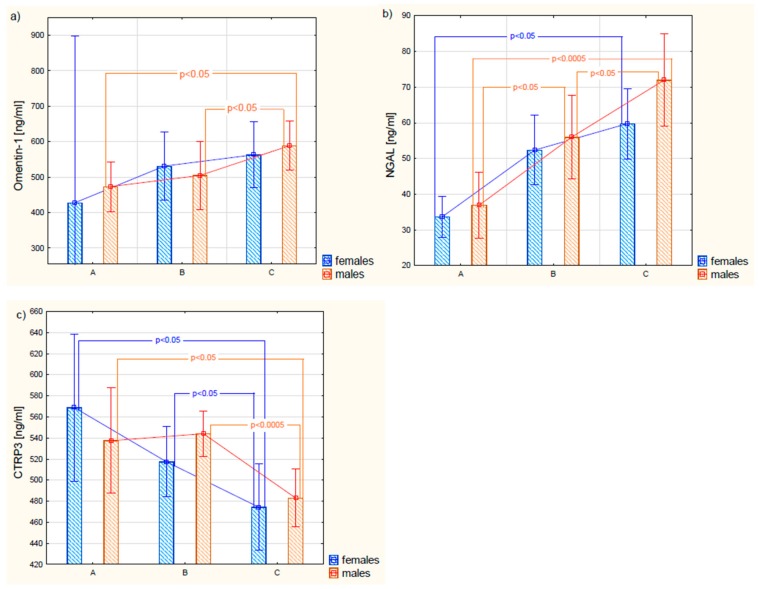
Gender differences in plasma omentin-1 (**a**), NGAL (**b**), and CTRP3 (**c**) levels in control subjects (A), and obese patients with type 2 diabetes before the implementation of insulin therapy (B), and in the same patients after 6-month treatment period (C). The results are expressed as mean ± 0.95 confidence interval.

As a result of the conducted research, it was proved that plasma concentrations of omentin-1 in patients with type 2 diabetes, before the implementation of insulin therapy, were not statistically significantly different from the concentration of this protein in the control group. In turn, the obtained results of omentin-1 in patients with type 2 diabetes after treatment with insulin were higher in relation to the values of plasma protein concentration before the implementation of therapy, as well as in comparison with people with metabolically healthy obesity. It was also shown that there were no statistically different plasma concentrations of omentin-1 between females and males within the same group. There were no significant differences in omentin-1 concentrations between females in all studied groups; however, in males with diabetes subjected to a 6-month treatment, omentin-1 plasma concentrations were significantly higher than those observed in both the control group and patients before therapy (Figure 1).

The conducted studies showed a significant positive correlation between the plasma concentration of omentin-1 and the HbA1c level (r = 0.358; *p* = 0.025) in T2DM after a 6-month insulin therapy. The significance of this relationship is difficult to explain, especially that omentin-1 increases insulin signal transduction by activating the protein kinase B and enhances insulin-mediated glucose transport in adipocytes. The obtained results may indicate complex and not fully explained mechanisms regulating the plasma concentration of analyzed adipokine.

For the next bioactive molecule analyzed in this work, i.e., NGAL, statistically significant differences between its plasma concentration in the obese control group and patients with type 2 diabetes were observed. In patients with DM, higher plasma NGAL concentrations were found than those in the control group. In addition, it was shown that during a 6-month treatment with a mixture of insulin, a further increase in this adipocytokine was observed. Statistically significant differences in the plasma concentration of NGAL occurred between men in the control group and men with type 2 diabetes. Moreover, statistically significant differences were found between NGAL concentrations in male T2DM patients prior to the implementation of insulin therapy in relation to this protein concentration in the same subjects after a 6-month insulin treatment period. In contrast, plasma NGAL levels in female patients who underwent a 6-month treatment were significantly higher than those determined before the treatment. However, there were no differences in plasma NGAL concentrations between women and men within the same study groups. The analysis of the relationship between the NGAL concentration and the BMI value showed a positive, strong correlation among control obese subjects (r = 0.56; *p* < 0.05). A weak negative correlation existed between NGAL and plasma fasting glucose (r = −0.35; *p* < 0.05) in diabetic patients qualified for insulin treatment as well as between NGAL and HDL (r = −0.40; *p* < 0.05) in patients after a 6-month therapy.

The conducted research also demonstrated that plasma CTRP3 levels in obese T2DM patients showed a downward trend in relation to the concentration of this molecule in controls. A statistically significant decrease in plasma CTRP3 concentration was observed in patients with DM treated with 6-month insulin therapy, in comparison to the values obtained in the same patients before the treatment was implemented. However, there were no differences in CTRP3 concentration in blood plasma between women and men within the same study group. Gender-related differences in circulating CTRP3 between groups were seen in women who underwent a 6-month insulin therapy, in comparison to CTRP3 levels found in these women before treatment, as well as in relation to the concentrations recorded in women from the control group. Similarly, in men with T2DM who underwent a 6-month insulin therapy, plasma CTRP3 levels were statistically significantly lower than in the group of patients qualified for the insulin treatment and the group of men from the control group. Moreover, there was a strong negative correlation between the plasma CTRP3 concentration and the BMI value in obese control subjects (r = −0.59; *p* < 0.05) as well as the average negative correlation between CTRP3 and total cholesterol (r = −0.31; *p* <0.05) and LDL (r = −0.35; *p* < 0.05) in patients qualified for treatment with an insulin mix. In the case of patients who underwent a 6-month therapy, the relationships between CTRP3 and BMI and carbohydrate-lipid metabolism parameters were not reported.

Moreover, as can be seen from Table 3, the analysis of the dependence between particular regulatory molecules showed that there was a positive, average correlation between serum concentration of NGAL and CTRP3 in the obese control group, whereas in T2DM patients qualified for insulin treatment, a positive correlation between the concentration of omentin-1 and CTRP3 was observed. However, no correlation was found between plasma levels of omentin-1 and NGAL in obese and diabetic patients, both qualified for insulin treatment and a 6-month insulin mixture therapy. In addition, there was no correlation between the concentration of omentin-1 and CTRP3 in the blood of obese patients and patients undergoing insulin mixture therapy, as well as between NGAL and CTRP3 levels in diabetic patients both before and after treatment.

## 4. Discussion

Although the majority of metabolic disorders in type 2 diabetes are attributable to impaired secretion and insulin action, contributing to hyperglycemia and the development of insulin resistance, there is much evidence to support the relationship between the development of insulin resistance and endocrine function of adipose tissue [17,18,19]. Adipocytokines secreted by adipose tissue participate in the pathogenesis of inflammatory processes, adipogenesis and regulation of energetic metabolism playing a crucial role in the pathomechanism of metabolic disorders such as obesity or type 2 diabetes [20]. The obtained results indicated that plasma omenting-1 levels in obese T2DM patients before implementation of insulin therapy were not statistically significantly different from the concentration of this regulatory molecule in the plasma of obese patients in the control group. In contrast to our results, other studies have reported decreased omentin-1 levels in both type 1 and type 2 diabetes [4,9,21,22] as well as in impaired glucose tolerance and obesity [23,24,25,26].

Many factors may affect the plasma profile of this molecule. Many authors point to an increased omentin-1 concentration in women’s blood plasma in relation to its plasma concentration in men’s blood [4,26]. The conducted research indicated that the omentin-1 concentrations in plasma were not statistically significantly different between women and men. Only in the case of men with type 2 diabetes subjected to a 6-month insulin mix therapy, plasma omentin-1 concentrations were significantly higher as compared to the observed concentration in the same men before implementation of the therapy as well as in the men from the control group. Studies have so far indicated complex and independent regulatory plasma mechanisms of this adipokine’s concentration [27]. A balanced and healthy diet with physical effort may lead to an increase of omentin-1 plasma concentration, which is simultaneously linked to the increase of tissues’ insulin sensitivity [28]. It is believed that omentin may have a protective action inhibiting the development of vascular complications [29,30]. Studies so far have revealed that the mention plasma concentration shows a relationship with the presence of late complications of macroangiopathic character developing in patients with type 2 diabetes [29]. The omentin concentration in patients with DM 2 with atherosclerotic plaque was statistically lower than in the group of patients with type 2 diabetes but without an atherosclerotic plaque. Moreover, in patients with diabetic nephropathy as well as diabetic retinopathy, the concentration of the protein in question was lower than in the group of patients with DM2 but without chronic kidney disease or retinopathy [30]. A key mechanism in the development of diabetic retinopathy is angiogenesis. As shown in the previous studies, omentin-1 may markedly decrease the expression of the vascular endothelial growth factor, which is associated with endothelial cells’ migration and angiogenesis within tiny blood vessels. The data suggest that omentin-1 may be a significant antiangiogenic factor playing an essential protective role in the development of microvascular disorders.

Apart from inhibiting the synthesis of adhesive molecules in endothelial cells, omentin-1 induces vascular relaxation dependent on nitrogen oxide. Moreover, it shows the capability of inhibiting the TNFα secretion by macrophages and cytokines inducing the inflammation in endothelial cells of blood vessels [30]. Taking into account the protective nature of omentin, the dynamic determination of its concentration in blood plasma in patients with type 2 diabetes may be helpful in the assessment of the effectiveness of the applied insulin therapy in delaying the development of vascular disorders, but further research is needed to confirm this relationship.

Implemented insulin mixture therapy was proved to be an important factor modifying the plasma profile of omentin-1, increasing the concentration of this bioactive protective molecule in the plasma of patients with type 2 diabetes, after 6 months of its use, in relation to pre-treatment situation. The results are difficult to compare with the literature data, since the plasma profile of omentin-1 expression in obese patients with type 2 diabetes subjected to an insulin mix therapy has not been assessed so far. However, recent studies have indicated that 6 months of treatment with metformin and liraglutide significantly increased plasma concentration of omentin-1 in patients with DM 2 [31]. Moreover, 6-month metformin therapy caused a marked increase of omentin-1 concentration in the blood of obese patients with insulin resistance in the course of PCOS [32]. A beneficial effect of pharmacotherapy confirmed both in the above-mentioned studies and in the studies being the subject of this paper may reflect the antidiabetogenic activity of omentin-1 in the compensation of metabolic disorders and its anti-inflammatory activity. Omentin-1, secreted mainly by visceral adipose tissue stromal cells, increases the transduction of the insulin signal by activating the protein kinase Akt/protein kinase B, and intensifies insulin-insulin-stimulated glucose transport in human adipocytes [22,33]. Unlike adiponectin, omentin-1 may inhibit the activation of JNK (c-Jun N-terminal kinase) cascade, activated by stress factors and singling transduction regulating the activity of many proteins, enzymes, and transcription factors as well as the one affecting inflammatory mechanisms [34]. Omentin-1 contributes to the regulation of lipids’ metabolism. In addition, it stimulates the AMPK activation, which has an inhibiting effect on the crucial lipolysis enzymes such as 3-hydroxy-3-methyl-glutaral—coenzymeA reductase (HMG-CoA) and acetyl CoA carboxylase (ACC). The AMPK activation favors oxidation of fatty acids and glucose uptake in myocytes, decreases gluconeogenesis in hepatocytes and inhibits lipogenesis in adipocytes [33]. Similar observations were also drawn by Yu and Herder et al. [35,36], who stated that omentin-1, by activating AMPK, plays a significant role in the energetic metabolism of the system. Additionally, it was observed that simultaneous insulin and omentin-1 therapy favorably influences the lipid profile which was reflected in the decrease of plasma cholesterol concentration, LDL cholesterol and triglycerides as well as in the increase of plasma HDL cholesterol concentration [33]. The above results indicate that apart from insulinomimetic activity, omentin-1 has a beneficial effect on the lipid metabolism disorders which constitute an independent risk factor for the development of DM type 2 complications, especially in obese people. In view of the above, the increase of plasma omentin-1 concentration in patients with type 2 diabetes after a six-month insulin treatment seems to be the result of beneficial influence of the applied therapy in terms of the compensation of metabolic disorders accompanying obesity and co-participating in the pathomechanism of the disease.

Moreover, the conducted research showed that in type 2 diabetes with obesity there is a change in adipose tissue metabolism, expressed by the elevated plasma concentration of NGAL, in relation to the concentration of this regulatory molecule in plasma of subjects with metabolically healthy obesity, which can confirm the role of this adipocytokine in the pathomechanism of obesity leading to the development of insulin resistance, and consequently type 2 diabetes. Recently, increasing evidence has suggested that NGAL not only plays a significant role in both glucose and lipid metabolism, but also modulates immune and inflammatory signaling, which is important in diabetes and metabolic syndrome [10]. The studies have so far indicated that the protein is also linked to the regulation of insulin resistance and development of diabetic vascular disorders, in particular diabetic nephropathy [7,37,38]. The obtained results are compatible with the results of other authors who also pointed out that NGAL plasma in people with impaired glucose tolerance and/or diabetes was higher than in the group of obese patients [39,40]. Moreover, the NGAL concentration directly correlated with obesity, hypecholesterolaemia and hyperglycaemia in patients with metabolic and cardiovascular diseases [40]. Both in those studies and ours the relationship between circulating NGAL with plasma fasting glucose in T2DM patients before implementation of insulin treatment as well as with HDL cholesterol in patients after a 6-month insulin therapy was found. Moreover, insulin mixture therapy was found as an important factor modifying the plasma profile of NGAL, increasing the concentration of this bioactive molecule in the plasma of patients with type 2 diabetes, after 6 months of its use, in relation to the concentration before treatment. Analyzing the differences in the NGAL plasma concentration by sex, it was found that only in the group of men the therapy with insulin mix led to an NGAL increase in the plasma in relation to patients before treatment. The above results indicate that the body’s response to treatment with insulin mix depends also on sex, which can partially be linked to a varied development of adipose tissue in women and men and the influence of sex hormones on the adipose tissue activity.

In relation to the last adipocytokine analyzed in this study, i.e., CTRP3, it was found that the CTRP3 plasma concentration in individuals with DM type 2, qualified for insulin mix therapy, was showing a decreasing trend when compared to the control group. However, a significant decrease of the adipocytokine in question in plasma of individuals with DM type 2 treated for the period of 6 months with an insulin mix was detected—both in relation to the control group and patients before starting the therapy. In addition, significant differences were found in the concentration of the mentioned protein between women after 6-month insulin therapy in relation to the value of CTRP3 observed in those women before implementing the therapy, as well as in relation to the concentration of the protein in question in plasma of women from the control group. Similar relationships were observed in relation to men. In most studies circulating CTRP3 levels were found higher in women than in men [41,42], with one exception [43]. However, the gender-specific regulation of CTRP3 still needs to be explored in more detail. Recent studies which assess the CTRP3 concentration in patients with diabetes are also contradictory. In Choi et al.’s study [44], an increase in the level of CTRP3 in patients with DM type 2 was found; however, other studies showed a decrease of the discussed adipocytokine in patients with DM type 2 and/or obesity [13,15,41,42,43], which corresponds with the results obtained in this research paper. Considering that CTRP3 is an anti-inflammatory mediator, being also negatively associated with the proinflammatory cytokines TNF, IL-6, and C-reactive protein [13,14,44] as well as enhancing glucose tolerance [8,15], we can conclude from the obtained results that the decrease of cartonectin concentration in blood plasma of patients subjected to a 6-month therapy with an insulin mix was associated with reducing inflammation and insulin resistance in these patients. On the other hand, it is difficult to clearly decide if the observed result is a consequence of pharmacotherapy or ongoing changes of endocrine adipose tissue’s activity in the range of CTRP3 synthesis as a modulating substance for insulin sensitivity and inflammation.

The conducted research allowed to find changes in metabolic homeostasis parameters in patients with type 2 diabetes after 6-month insulin therapy. The insulin mixture treatment influenced the increase in HbA1c, triglyceride and uric acid, but exhibited no effect on the levels of other metabolic homeostasis parameters. An extended period of observation on a larger sample size could result in more favorable outcomes with regard to long-term glycemic control and the improvement of the lipid profile.

To the best of our knowledge, plasma profile of omentin-1, NGAL, and CTRP3 has not been evaluated as yet in obese type 2 diabetic patients during insulin mixture therapy. Despite quite numerous publications regarding the role of analyzed adipocytokines in type 2 diabetes, there are no data investigating the effect of insulin mixture therapy on plasma levels of these molecules. Moreover, the initiation of insulin therapy with insulin mixture formulations is an infrequently chosen therapeutical option in type 2 diabetic patients, which was the reason for difficulties in collecting the appropriate, highly selected group of patients. Additional large-scale studies are needed to further explore the role of biologically active molecules such as C1q/TNF-related protein 3, omentin-1 and NGAL as well as the importance of their impaired plasma profile before being considered as a routine clinical option in monitoring metabolic changes in T2DM patients.

## 5. Conclusions

In conclusion, the significant changes in the plasma profile of all biologically active substances evaluated in our study, which are secreted by the adipose tissue, i.e., omentin-1, NGAL, and CTRP3, suggest their potential diagnostic utility in monitoring metabolic changes associated with the introduction of insulin treatment in type 2 diabetic patients. Moreover, the applied therapy led to a different nature of changes in plasma concentrations of adipocytokines with anti-inflammatory activity, i.e., an increase in omentin-1 concentration and a decrease in CTRP3 concentration, reflecting the complex nature of the endocrine activity of fat tissue in regulating the inflammatory process associated with type 2 diabetes.

## Figures and Tables

**Table 1:** Clinical characteristics of control subjects and obese patients with type 2 diabetes mellitus.

Parameters	Control Subjects	Obese Patients with Type 2 Diabetes Mellitus	
		Before the Implementation of Insulin Therapy	After a 6-Month Insulin Treatment
**Gender Female/Male (*n*)**	20 (7F/13 M)	42 (19 F/23 M)	42 (19 F/23 M)
Age (yr)	54.81 ± 8.64	60.98 ± 5.63	61.76 ± 6.63
Disease duration (yr)	–	13.05 ± 6.49	13.65 ± 6.49
BMI (kg/m^2^)	29.63 ± 5.40	33.09 ± 4.09	33.03 ± 4.14
WHR (cm^2^)	0.95 ± 0.07	1.01 ± 0.05	1.00 ± 0.06
Glucose (mg/dL)	91.38 ± 6.86	134.12 ± 30.03 ^a^	136.64 ± 25.53
HbA1c (%)	5.19 ± 0.34	7.30 ± 1.10 ^a^	7.67 ± 1.03 ^b^
Triglycerides (mg/dL)	168.95 ± 59.46	163.63 ± 88.11	179.15 ± 69.33 ^b^
Cholesterol (mg/dL)	193.31 ± 34.77	183.75 ± 55.08	186.93 ± 30.01
HDL (mg/dL)	46.99 ± 8.90	50.92 ± 16.62	49.01 ± 14.59
LDL (mg/dL)	116.03 ± 29.12	107.49 ± 36.70	100.79 ± 28.17
Creatinine (mg/dL)	0.84 ± 0.09	0.87 ± 0.23	0.92 ± 0.35
Uric acid (mg/dL)	5.15 ± 0.79	5.53 ± 1.56	7.17 ± 1.44 ^b^
ALT (U/L)	29.75 ± 8.02	30.92 ± 16.12	34.12 ± 14.36
TSH (mU/L)	2.43 ± 0.33	1.88 ± 1.79	1.45 ± 0.43
CRP (mg/L)	0.85 ± 0.58	0.69 ± 0.51	0.23 ± 0.15

Values given as mean ± SD; F, female; M, male; BMI, body mass index; WHR, waist–hip ratio; HbA1c, glycated haemoglobin; HDL, high density lipoprotein; LDL, high density lipoprotein; ALT, alanine aminotransferase; TSH, thyroid stimulating hormone CRP, C-reactive protein; ^a^ *p* < 0.0001 compared with controls; ^b^ *p* < 0.05 compared with obese patients with T2DM before insulin therapy.

**Table 2:** Concentrations of plasma omentin-1, NGAL and CTRP3 in control subjects and obese patients with type 2 diabetes mellitus.

Parameters	Control Subjects	Obese Patients with Type 2 Diabetes Mellitus	
		Before the Implementation of Insulin Therapy	After a 6-Month Insulin Treatment
Omentin-1 (ng/mL)	464.41 ± 214.92	516.44 ± 209.54	576.88 ± 174.31 ^a,d^
NGAL (ng/mL)	36.98 ± 14.76	54.37 ± 23.24 ^b^	66.51 ± 25.76 ^c, e^
CTRP3 (ng/mL)	543.71 ± 69.86	531.98 ± 60.1	479.06 ± 72.95 ^b, f^

Values given as mean ± SD; NGAL, neutrophil gelatinase-associated lipocalin; CTR3, C1q/TNF-related protein-3; ^a^ *p*<0.05, compared to controls; ^b^ *p* <0.01, compared to controls; ^c^ *p* <0.0001, compared to controls; ^d^ *p* <0.05, compared to diabetic patients before insulin therapy; ^e^ *p* <0.01, compared to diabetic patients before insulin therapy; ^f^ *p* <0.0001, compared to diabetic patients before insulin therapy.

**Table 3:** Correlation between plasma concentration of the adipocytokines and the body mass index, and carbohydrate-lipid metabolism parameters in obese patients with type 2 diabetes before the implementation of insulin therapy and after 6-month treatment.

**Parameters**	**Obese Patients with Type 2 Diabetes Mellitus Before the Implementation of Insulin Therapy**		
	Omentin-1 (ng/mL)	NGAL (ng/mL)	CTRP3 (ng/mL)
BMI (kg/m^2^)	−0.060 ^NS^	−0.114 ^NS^	0.137 ^NS^
Glucose (mg/dL)	0.135 ^NS^	−0.345 (*p* = 0.032)	−0.058 ^NS^
HbA1c (%)	0.158 ^NS^	−0.016 ^NS^	−0.129 ^NS^
Cholesterol (mg/dL)	0.140 ^NS^	−0.020 ^NS^	−0.305 (*p* = 0.048)
HDL (mg/dL)	−0.092 ^NS^	−0.211 ^NS^	−0.229 ^NS^
LDL (mg/dL)	0.145 ^NS^	0.039 ^NS^	−0.345 (*p* = 0.033)
Triglycerides (mg/dL)	−0.076 ^NS^	−0.019 ^NS^	−0.015 ^NS^
NGAL (ng/mL)	0.029 ^NS^		
CTRP3 (ng/mL)	0.354 (*p* = 0.024)	0.04 ^NS^	
	**Obese Patients with Type 2 Diabetes Mellitus After the 6-Month Insulin Treatment**		
	Omentin-1(ng/mL)	NGAL (ng/mL)	CTRP3 (ng/mL)
BMI (kg/m^2^)	0.021 ^NS^	−0.083 ^NS^	0.073 ^NS^
Glucose (mg/dL)	0.200 ^NS^	−0.082 ^NS^	0.033 ^NS^
HbA1c (%)	0.358 (*p* = 0.025)	0.082 ^NS^	−0.192 ^NS^
Cholesterol (mg/dL)	0.030 ^NS^	0.072 ^NS^	0.032 ^NS^
HDL (mg/dL)	−0.072 ^NS^	−0.396 (*p* = 0.041)	−0.021 ^NS^
LDL (mg/dL)	0.096 ^NS^	0.175 ^NS^	−0.069 ^NS^
Triglycerides (mg/dL)	−0.055 ^NS^	0.107 ^NS^	−0.262 ^NS^
NGAL (ng/mL)	−0.003 ^NS^		
CTRP3 (ng/mL)	0.16 ^NS^	−0.25 ^NS^	

## Abbreviations

ACC	acetyl-CoA carboxylase
ADPN	adiponectin
BMI	body mass index
CTRP3	complement C1q tumor necrosis factor-related protein-3
HMG-CoA	3-hydroxy-3-methylglutaryl coenzyme A
NGAL	neutrophil gelatinase-associated lipocalin
T2DM	type 2 diabetes mellitus
VEGF	vascular endothelial growth factor
WHR	waist-hip ratio
